# Central-to-peripheral blood pressure amplification: role of the recording site, technology, analysis approach, and calibration scheme in invasive and non-invasive data agreement

**DOI:** 10.3389/fcvm.2023.1256221

**Published:** 2023-10-11

**Authors:** Yanina Zócalo, Daniel Bia, Ramiro Sánchez, Gustavo Lev, Oscar Mendiz, Agustín Ramirez, Edmundo I. Cabrera-Fischer

**Affiliations:** ^1^ Departamento de Fisiología, Centro Universitario de Investigación, Innovación y Diagnóstico Arterial (CUiiDARTE), Facultad de Medicina, Universidad de la República, Montevideo, Uruguay; ^2^ Metabolic Unit and Hypertension Unit, University Hospital, Favaloro Foundation, Buenos Aires, Argentina; ^3^ Department of Interventional Cardiology, University Hospital, Favaloro Foundation, Buenos Aires, Argentina; ^4^ Instituto de Medicina Traslacional, Trasplante y Bioingeniería (IMETTYB), Favaloro University—CONICET, Buenos Aires, Argentina

**Keywords:** applanation tonometry, calibration, central-to-peripheral blood pressure amplification, invasive records, non-invasive records, oscillometry, physiology, vascular ultrasound

## Abstract

**Background:**

Systolic blood pressure amplification (SBPA) and pulse pressure amplification (PPA) can independently predict cardiovascular damage and mortality. A wide range of methods are used for the non-invasive estimation of SBPA and PPA. The most accurate non-invasive method for obtaining SBPA and/or PPA remains unknown.

**Aim:**

This study aims to evaluate the agreement between the SBPA and PPA values that are invasively and non-invasively obtained using different (1) measurement sites (radial, brachial, carotid), (2) measuring techniques (tonometry, oscillometry/plethysmography, ultrasound), (3) pulse waveform analysis approaches, and (4) calibration methods [systo-diastolic vs. approaches using brachial diastolic and mean blood pressure (BP)], with the latter calculated using different equations or measured by oscillometry.

**Methods:**

Invasive aortic and brachial pressure (catheterism) and non-invasive aortic and peripheral (brachial, radial) BP were simultaneously obtained from 34 subjects using different methodologies, analysis methods, measuring sites, and calibration methods. SBPA and PPA were quantified. Concordance correlation and the Bland–Altman analysis were performed.

**Results:**

(1) In general, SBPA and PPA levels obtained with non-invasive approaches were not associated with those recorded invasively. (2) The different non-invasive approaches led to (extremely) dissimilar results. In general, non-invasive measurements underestimated SBPA and PPA; the higher the invasive SBPA (or PPA), the greater the underestimation. (3) None of the calibration schemes, which considered non-invasive brachial BP to estimate SBPA or PPA, were better than the others. (4) SBPA and PPA levels obtained from radial artery waveform analysis (tonometry) (5) and common carotid artery ultrasound recordings and brachial artery waveform analysis, respectively, minimized the mean errors.

**Conclusions:**

Overall, the findings showed that (i) SBPA and PPA indices are not “synonymous” and (ii) non-invasive approaches would fail to accurately determine invasive SBPA or PPA levels, regardless of the recording site, analysis, and calibration methods. Non-invasive measurements generally underestimated SBPA and PPA, and the higher the invasive SBPA or PPA, the higher the underestimation. There was not a calibration scheme better than the others. Consequently, our study emphasizes the strong need to be critical of measurement techniques, to have methodological transparency, and to have expert consensus for non-invasive assessment of SBPA and PPA.

## 1. Introduction

The majority of studies aimed at evaluating the relationship between blood pressure (BP) and physiological or clinical conditions have mostly focused on brachial artery (BA) systolic, diastolic, or pulse pressure (bSBP, bDBP, or bPP, respectively). This could be because, on the one hand, brachial BP (bBP) parameters were found to be markers of cardiovascular risk, morbidity, and mortality, and, on the other hand, bBP was considered to accurately reflect the hemodynamic conditions of the arterial system as a whole (1). However, central aortic BP (aoBP) has gained growing interest and attention over the last few years. It has been proposed that the central one would show higher levels of association with cardiac or central arterial properties compared to bBP and aoBP would be more closely associated with the individual cardiovascular risk (2–4). The positing of an “opposition between bBP and aoBP,” and the “need” to define the superiority of one over the other, does not allow us to consider that the (relative) higher usefulness could depend on the context and to consider the possibility of complementarity and/or that the integration of the information they provide could be the most useful. On the other hand (and in relation to the preceding), understanding the relationship between bBP and aoBP would allow us to obtain information on both the vascular system “as a whole” and “local” hemodynamic conditions from the data acquired at a single recording site (e.g., the brachial). In this regard, it is known that with the subject at supine rest, diastolic BP (DBP) and mean BP (MBP) remain relatively constant among central and peripheral arteries, whereas systolic blood pressure (SBP) and pulse pressure (PP) are expected to be higher in the latter (5–8). This systolic blood pressure amplification (SBPA) or pulse pressure amplification (PPA) depends (among other factors) on the distance between the arterial sites, the stiffness gradient, and the timing and magnitude of the wave reflections (8–11). Several studies showed that SBPA and PPA would be independently associated with target organ damage and cardiovascular risk (12–16). Furthermore, compared to aoBP and bBP alone, PPA would be superior as a cardiovascular risk predictor in hypertension and kidney disease (17). In addition, lower SBPA or PPA (for a given MBP) in adults would be associated with worse hemodynamic conditions for the heart and central arteries (7). In turn, we recently found that SBPA and PPA levels would be associated with structural cardiac properties at early ages in life (e.g., in children) (18).

SBPA or PPA can be determined from non-invasive bBP and aoBP estimation. Non-invasive bBP measurement methods are well defined and established; however, up to now, there is no consensus on which (if any) would be the best approach to non-invasively determine aoBP (and subsequently quantify SBPA or PPA) (19, 20). aoBP can be determined using approaches that differ in the technical principles used to record pulse waveform or surrogate signals, e.g., in the recording site [common carotid artery (CCA), radial artery (RA), BA] and/or in the algorithm or mathematical method applied (19–22). The abovementioned differences could result in significant differences in aoSBP and aoPP and, consequently, in SBPA and PPA (19, 20). On the other hand, when the recorded pulse waves are calibrated to non-invasive bBP (as is usually done), the agreement between invasive and non-invasive aoBP data and, consequently, between invasive and non-invasive SBPA and PPA levels could depend on the “calibration scheme or method” being considered (23–28). In this regard, the most commonly used calibration schemes calibrate the pulse waveforms using either the bSBP and bDBP [“systo-diastolic calibration” (SD)] or the bDBP and brachial MBP (bMBP) (3, 18, 19). In turn, the bMBP used for calibration could be obtained (measured) by oscillometry (“osc” calibration) or calculated from bSBP and bDBP, by applying different equations (e.g., a form factor equal to 33% or 41.2%) (19, 29, 30). In this scenario, it remains to be determined whether the differences between invasive and non-invasive SBPA (or PPA) levels are modified by the calibration method and/or the mathematical approach used to derive the bMBP.

This study aimed to evaluate the association and agreement between SBPA and PPA values invasively and non-invasively obtained considering different:

(i) recording sites (CCA, BA, and RA),
(ii) recording techniques (oscillometry/plethysmography, tonometry, ultrasound),
(iii) analyses (e.g., direct and indirect analysis of the pulse waveform),
(iv) mathematical approaches to estimate bMBP, and
(v) calibration methods.

Complementing previous work, in which only invasive aortic recordings were performed, we performed invasive aortic and brachial recordings in the present study as a way to quantify central-to-peripheral BP amplification (SBPA and PPA) and compare it with multiple non-invasive approaches. Then, it could be considered as a (natural) continuation of a recently published work (20).

## 2. Materials and methods

### 2.1. Group assessment

The study involved subjects (*n* = 34) undergoing coordinated angiography of the coronary vessels at the University Hospital (Favaloro Foundation, Buenos Aires, Argentina) (20, 31). A recent study of our group that focused on different hemodynamic issues used data from the same population (20). The exclusion criteria included heart valve diseases and cardiac arrhythmias. Prior to the evaluation, written informed consent was obtained from the subjects or their parents. If applicable, assent was also obtained. The protocol was approved by the Institutional Ethics Committee, and all procedures agreed with the Declaration of Helsinki.

The exposure of the subjects to cardiovascular risk factors (defined as previously described and following international recommendations) was determined from a clinical interview, biochemical analysis, echocardiographic studies, and anthropometric evaluation (32–36) (Table 1).

**Table 1:** Demographic, anthropometric, and clinical characteristics of the studied subjects.

Variable	MV	SE	SD	Min.	p25th	p50th	p75th	Max.	Range
Age (years)	61	3	19	14	52	68	72	89	75
Body weight (kg)	75.5	2.6	15.3	46	65	73	88	103	57
Body height (m)	166	2	9	147	162	165	174	182	35
BMI (kg/m^2^)	27.2	0.8	4.4	17.5	24.2	27.0	29.9	38.9	21.3
Hemoglobin (g/L)	12.5	1.2	2.6	8.4	12.3	12.8	13.9	15.3	6.9
Hematocrit (%)	38.1	1.8	6.6	26.0	36.0	38.3	43.0	46.0	20.0
Total cholesterol (mg/dL)	184.0	22.5	59.5	122.0	131.0	170.0	239.0	287.0	165.0
HDL cholesterol (mg/dL)	45.7	5.1	13.5	29.0	39.0	41.0	54.0	71.0	42.0
LDL cholesterol (mg/dL)	114.7	23.7	62.6	64.0	66.0	78.0	186.0	218.0	154.0
Triglycerides (mg/dL)	119.1	22.8	60.2	69.0	71.0	103.0	151.0	238.0	169.0
Atherogenic index	4.45	0.89	2.36	2.56	2.85	3.05	7.36	8.24	5.68
Creatinine (mg/dL)	1.15	0.10	0.36	0.79	0.80	1.07	1.46	1.90	1.11
Urea (mg/dL)	50.0	6.6	22.9	28.0	33.0	38.5	66.0	99.0	71.0
Glycemia (mg/dL)	109.4	14.0	41.9	74.0	89.0	90.0	106.0	197.0	123.0
Sodium(mEq/L)	132.7	1.5	4.6	122.0	132.0	133.0	136.0	137.0	15.0
Potassium (mEq/L)	4.1	0.2	0.5	3.4	3.7	4.3	4.6	4.7	1.3
LV EDD (mm)	52.3	2.9	10.4	38.0	42.0	53.0	59.0	71.0	33.0
LV ESD (mm)	31.2	2.5	8.8	18.0	25.0	30.5	37.5	45.0	27.0
LV septum thickness mm	10.5	0.7	2.4	6.8	8.0	11.6	12.0	14.0	7.2
LV posterior wall thickness mm	9.2	0.6	2.0	6.5	7.0	9.0	11.0	12.0	5.5
Left atrium area (cm^2^)	26.5	2.1	5.1	18.0	25.0	26.5	30.0	33.0	15.0
LV ejection fraction (%)	59	2	8	38	55	60	65	70	32
Active smokers (%)	5.9								
Ex-smokers (%)	48.3								
Arterial hypertension (%)	69.7								
Diabetes (%)	30.3								
Diabetics requiring insulin (%)	25.0								
Dyslipidemia (%)	60.6								
Renal insufficiency (%)	18.2								
Myocardial infarction (%)	18.2								
Acute coronary syndrome (%)	7.4								
CABG (%)	12.1								
Coronary angioplasty (%)	15.2								
ACEI (%)	37.5								
ARBs (%)	29.2								
MRAs (%)	12.5								
Beta-blockers (%)	50.0								
Diuretics (%)	20.8								
Calcium channel blockers (%)	29.2								
Antiplatelet therapy (%)	31.3								
Statins (%)	66.7								
T4 (%)	8.3								

MV, mean value; SE and SD, standard error and deviation, respectively; Min. and Max., minimum and maximum value, respectively; p25th, p50th, and p75th, percentiles 25th, 50th, and 75th, respectively; BMI, body mass index; LV, left ventricle; EDD and ESD, end-diastolic and end-systolic diameter, respectively; CABG, coronary artery bypass graft surgery; ACEI, angiotensin-converting enzyme inhibitors; ARBs, angiotensin II receptor blockers; MRAs, mineralocorticoid receptor antagonists; LDL, low-density lipoprotein; HDL, high-density lipoprotein.

**Figure 1:**
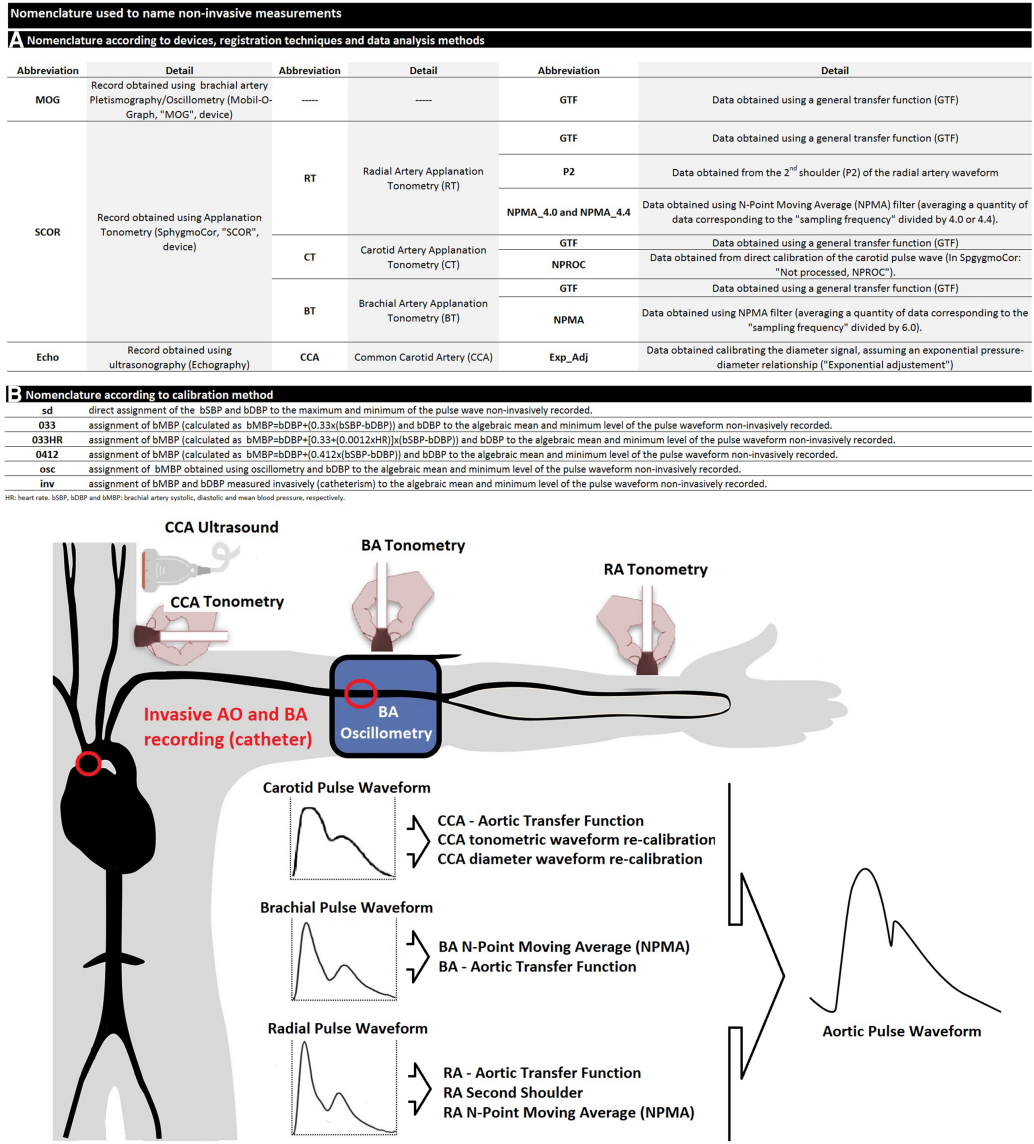
(**A**) nomenclature used to designate non-invasive-derived variables. (**B**) schematic representation of invasive and non-invasive blood pressure (BP) measurements in the aortic root and brachial artery. AO, aorta; CCA, common carotid artery; BA, brachial artery; RA, radial artery.

Invasive (catheterism) aoBP and bBP levels and waveforms were measured. In addition, aoBP levels and waveforms were non-invasively obtained from:

(i) CCA, BA, and RA applanation tonometry recordings (SphygmoCor device SCOR, v.9, AtCor Medical, Sydney, NSW, Australia),
(ii) BA oscillometric/plethysmographic measurements [Mobil-O-Graph (MOG), Model PWA, IEM GmbH, Stolberg, Germany], and
(iii) CCA vascular ultrasound (6–13 MHz, M-Turbo, Sonosite Inc., Bothell, WA, USA) (Figure 1).

Non-invasive data were obtained in random order to avoid potential bias associated with time intervals between non-invasive and invasive recordings.

### 2.2. Invasive BP recordings

While the subject was tested lying down on a catheterization table, intra-arterial aoBP and bBP levels and waveforms were recorded. After standard asepsis of the RA access area, lidocaine was subcutaneously injected, whereas soft sedation (1.5 mg of midazolam and 0.025 mg of fentanyl) was administered to minimize pain and discomfort. An intra-arterial introducer sheath was placed, and heparin was infused. A 0.035 in guide wire was moved forward and placed in the ascending aorta. Then a 5 French pigtail catheter (Cordis, Miami, USA) was introduced and positioned so that its tip remained always −4 cm away from the heart valve. The correct positioning of the catheter was confirmed (fluoroscopy, Allura Xper FD10 or Allura Clarity FD20/10, Philips Healthcare, Netherlands), and then the guide wire was removed, and the catheter was flushed with saline solution.

The fluid-filled catheter placed in the arterial lumen (aorta or BA) was connected to the external BP transducer (MX960, Medex, LogiCal, Smiths Medical ASD Inc., Minneapolis, USA) coupled to the Acist CVi system (Acist CVi, Medical System Inc., Germany). In turn, the Asist CVi system was synchronized with the x-ray imaging system Allura Xper FD10 or Allura Clarity FD20/10. The MX960 meets (or surpasses) the specifications of the Association for the Advancement of Medical Instrumentation/European Society of Hypertension/International Organization for Standardization (AAMI/ESH/ISO) collaboration.

The system (catheter, tubing, and external transducer) was flushed with saline solution, and the arterial pressure (aoBP or bBP) waveform was visually inspected for quality before each recording. The external transducer was calibrated following the built-in two-point calibration method of the system. First, the zero was assigned to the recording obtained with the sensor opened to the atmosphere (adjusting the baseline to zero or atmospheric pressure), and the second point was obtained by exposing the transducer to 100 mmHg (done by the device itself). The dynamic response of the system was adjusted to ensure a natural frequency of at least 20 Hz and a damping coefficient of at least 0.3. External transducers similar to the ones used in the present work (MX960, LogiCal) have high-quality, distortion-free frequency responses within the bandwidth of 0–30 Hz (37). This guarantees an adequate arrangement between the natural frequency and damping coefficient, ensuring that the measurement systems operate in areas of adequate dynamic responses (or in a somewhat underdamped region). The external transducer was always maintained at the cardiac level (midaxillary line). BP waveforms were visualized in the Allura Xper FD10 or Allura Clarity FD20/10 monitor.

Simultaneously, aoBP was invasively obtained and estimated using non-invasive ultrasound, oscillometry/plethysmography, and applanation tonometry recordings (see below) (Figure 1).

After recording aoBP, the catheter was moved to the BA opposite to that of the vascular access and positioned at the level where the pneumatic BA cuff for non-invasive BP measurement (MOG device) was located. Intra-arterial BP levels and waveforms were then measured, and non-invasive BP data were obtained (immediately before or after) using the oscillometric/plethysmographic technique (MOG device) (see below). After each bBP recording, the catheter was repositioned in the ascending aorta, and aoBP levels and waveforms were recorded, enabling hemodynamic stability to be confirmed.

The invasive recordings and data processing systems were used to determine heart rate (HR) and systolic, diastolic, and mean (i.e., from the pressure/time curve integral) BP levels.

After the evaluations, the subject was sent to the recovery area and discharged from the university hospital as appropriate considering his/her clinical situation.

### 2.3. Non-invasive BP recordings and determination of the mean BP

At the same time, immediately before and/or after each invasive recording, bBP levels and waveforms were recorded by oscillometry/plethysmography (MOG device) through a pneumatic cuff positioned in the arm contralateral to the used for the vascular access (26, 38, 39). Non-invasive bBP values were used to calibrate CCA (vascular ultrasound), BA (applanation tonometry and oscillometry/plethysmography), and RA (applanation tonometry) pulse waveforms (see Figure 1).

The suffix “osc” was used to design data obtained with the oscillometric system (e.g., bSBPosc, bDBPosc, bPPosc, and HRosc). bMBP was directly obtained with oscillometry (bMBPosc). In addition, it was calculated from bSBPosc and bDBPosc considering two different form factors (33% and 41.2%), representing the percentage of the waveform amplitude added to the minimum (bDBPosc) to obtain the mean (bMBP) (20, 30):

(i) bMBP_0.412_ mmHg = bDBPosc + 0.412 × bPPosc
(ii) bMBP_0.33HR_ mmHg = bDBPosc + 0.33 + (0.0012 × HRosc) × bPPosc
(iii) bMBP_0.33_ mmHg = bDBPosc + 0.33 × bPPosc

The bMBP values obtained as stated above were used to calibrate the ultrasound-, tonometry-, and oscillometry/plethysmography-derived recordings used to obtain aoBP (Figure 1).

### 2.4. Central aoBP non-invasive estimation

#### 2.4.1. BA oscillometry/plethysmography recordings

We estimated the aoBP levels and waveforms estimated from the BA oscillometry/plethysmography recordings (MOG device) by applying a generalized transfer function (GTF). Only high-quality index (1 or 2) and satisfactory (visual inspection) BA pulse waveforms were considered. The device determined bBP (first inflation) and aoBP (second inflation) during the same “double” inflation-deflation cycle of the brachial cuff. The device was used to determine aoBP, bSBPosc, bMBPosc, and bDBPosc values used in its own calibration and in the calibration of tonometry and ultrasound-derived waveforms. Each aoBP data derived from MOG recordings was obtained by calibrating to (i) bSBPosc and bDBPosc (SD approach), (ii) bDBPosc and bMBPosc, and (iii) bDBPosc and bMBP_0.33_. Calibrating MOG records using invasive bBP levels (catheterism-derived) or other forms of estimating bMBP (i.e., bMBP_0.33HR_ and bMBP_0.412_) was not possible, because the device does not allow it. Unlike other devices (e.g., SphygmoCor), this device does not allow the clinician and/or researcher to calibrate in the way one wishes, limiting the possibility of investigating different calibration ways.

#### 2.4.2. CCA ultrasound recordings

Left CCAs were displayed in a longitudinal view (centimeter proximal to the carotid bulb) using ultrasound (Sonosite device). Videos (duration of ≥30 s, B-mode, longitudinal views) were recorded and stored for the off-line analysis that enabled us to obtain the beat-to-beat diameter waveforms using border detection software (Hemoydin4M software, Dinap, Buenos Aires, Argentina). In addition, aoBP waveform and levels were obtained from the diameter data (40–42). To this end, CCA diameter waveforms were calibrated using an exponential calibration scheme as in previous works (20, 40), applying a method that assumes an exponential pressure–diameter relationship:

$p(t)=p_{d}\exp\;\;\left[\alpha \left(\frac{A(t)}{\mathrm{Ad}}-\;1\right)\right]$, with $A(t)=\;\frac{\pi d^{2}(t)}{4}$, and $\alpha =\frac{\mathrm{Ad}\;\left(\mathrm{In}\frac{\mathrm{SBP}}{\mathrm{DBP}}\right)}{\mathrm{As}-\mathrm{Ad}}$,

where *p*(*t*) is the pressure; *d*(*t*) is the diameter; *A*(*t*) is the arterial cross-section as a function of time; Ad and As are end-diastolic and peak systolic cross-section area, respectively; and *α* is the BP-independent wall stiffness coefficient (42). Assuming that DBP and MBP remain constant throughout large and medium arteries when the subject is lying down, the iterative scheme could be used to determine *α* based on bMBP and bDBP.

To this end, (i) invasive bDBP and bMBP and (ii) bDBPosc and bMBP levels were used to calibrate CCA diameter waveforms. Specifically, CCA ultrasound-derived aoSBP and aoPP were obtained using four calibration schemes that included bDBPosc in conjunction with (i) bMBPosc and bMBPcalc, (ii) bMBP_0.33_, (iii) bMBP_0.33HR_, and (iv) bMBP_0.412_.

#### 2.4.3. CCA tonometry (CT) recordings

Central aoBP waveforms and levels were obtained using tonometry (SCOR device) applied to CCA, RA, and BA (random order) (Figure 1). Arterial tonometry provides the instantaneous BP waveform that can be scaled using different calibration methods. Only high-quality recordings (operator index >75%) and accurate waveforms (visual inspection) were analyzed.

From the carotid tonometry recordings (Figure 1), we obtained aoBP levels by (i) applying a carotid-to-aorta GTF (GTF approach) and (ii) without using a GTF (not-processed NPROC approach), considering carotid and aortic pulse waveforms as very similar due to the anatomical proximity (21, 43). Regardless of the method considered to obtain aoBP levels from carotid tonometry-derived data (as well as from BA and RA), pulse waveforms were calibrated to (i) invasively derived bDBP and bMBP and non-invasively derived, (ii) bSBPosc and bDBPosc, and (iii) bDBPosc and bMBP, using different ways to quantify bMBP, i.e., bMBPosc, bMBP_0.33_, bMBP_0.33HR_, and bMBP_0.412_.

#### 2.4.4. RA tonometry (RT) recordings

Central aorta BP waveforms and/or levels were obtained from radial tonometry-derived waveforms, considering four different data analysis methods.

First, aoBP levels and waveform were determined through a transfer function (radial-aortic GTF) (20, 34, 38). Second, aoSBP and aoPP were quantified from the second shoulder of the RA waveform (20, 44–46). Third and fourth, we quantified aoSBP and aoPP by applying a first-order low-pass filter (20). In this case, each single point in the recorded signal (RA or BA waveform) was summed up with its neighbors, and the result was divided by the number of points considered. The more points are averaged, the smoother the waveform obtained. The method was entitled “N-point moving average” (NPMA) (47). Because of its filtering characteristics, the NPMA method provides aoSBP levels and enables one to obtain aoPP, but in contrast to other methods, no information about aoBP waveforms is given (48). NPMA was initially proposed as a simple method to estimate aoSBP from RA-derived BP (47). Then, it was proposed it could be also used to analyze BA-derived BP waves (49). The number of averaged points (*N*) differs depending on the BP waves (RA or BA) considered: *N* = Fs/4 (47) or *N* = Fs/4.4 (50) for RA-derived waveforms and *N* = Fs/6 for waveforms obtained from the BA recordings (49) (Fs, sampling frequency; numbers 4.0, 4.4, and 6 represent the optimal integer denominator *K*). After the method was proposed by the original authors, Xiao et al. reported that *K* = 4.4 would estimate aoSBP more accurately than *K* = 4.0 (50). Then, when analyzing RA tonometry recordings in this work, the NPMA method was applied using both, *K* = 4.0 and *K* = 4.4. Using the SCOR device (Fs = 128 Hz), 32 (128/4) and 29 (128/4.4) points were averaged when considering RA waves. In turn, 21 (128/6) points were considered for BA (see below) (20).

#### 2.4.5. BA tonometry recordings

From BA waveforms obtained from tonometry recordings and calibrated using different schemes, we quantified aoBP by applying a GTF and the NPMA method (*k* = 6) (20, 49).

### 2.5. Methods for pulse waveform calibration: carotid, radial, and BA records

Non-invasive aoBP estimation involves three independent processes: (i) acquisition of a peripheral (e.g., CCA, RA, and BA) waveform, measured in voltage (e.g., mV) or mm; (ii) calibration with BP, to “convert” the waveform to one with pressure units (mmHg) instead of mm or mV; and (iii) estimation of aoBP (usually) via “mathematical filtering” of the recalibrated waveforms (22). In this work, calibration was done as follows.

(1) Invasive-derived (“Inv”): measured bMBP and bDBP were assigned, respectively, to the algebraic mean and minimum of the peripheral waveforms.
(2) Systo-diastolic (“SD”): bSBPosc and bDBPosc were assigned, respectively, to the maximum and minimum of the peripheral waveforms.
(3) Oscillometric-derived (“osc”): bMBPosc and bDBPosc were assigned, respectively, to the algebraic mean and minimum of the peripheral waves.
(4) Calculated MBP: bMBP (bMBP_033_, bMBP_033HR_, and bMBP_0412_) and bDBPosc were assigned, respectively, to the algebraic mean and minimum of the peripheral waveforms.

### 2.6. SBPA and PPA

**Figure 2:**
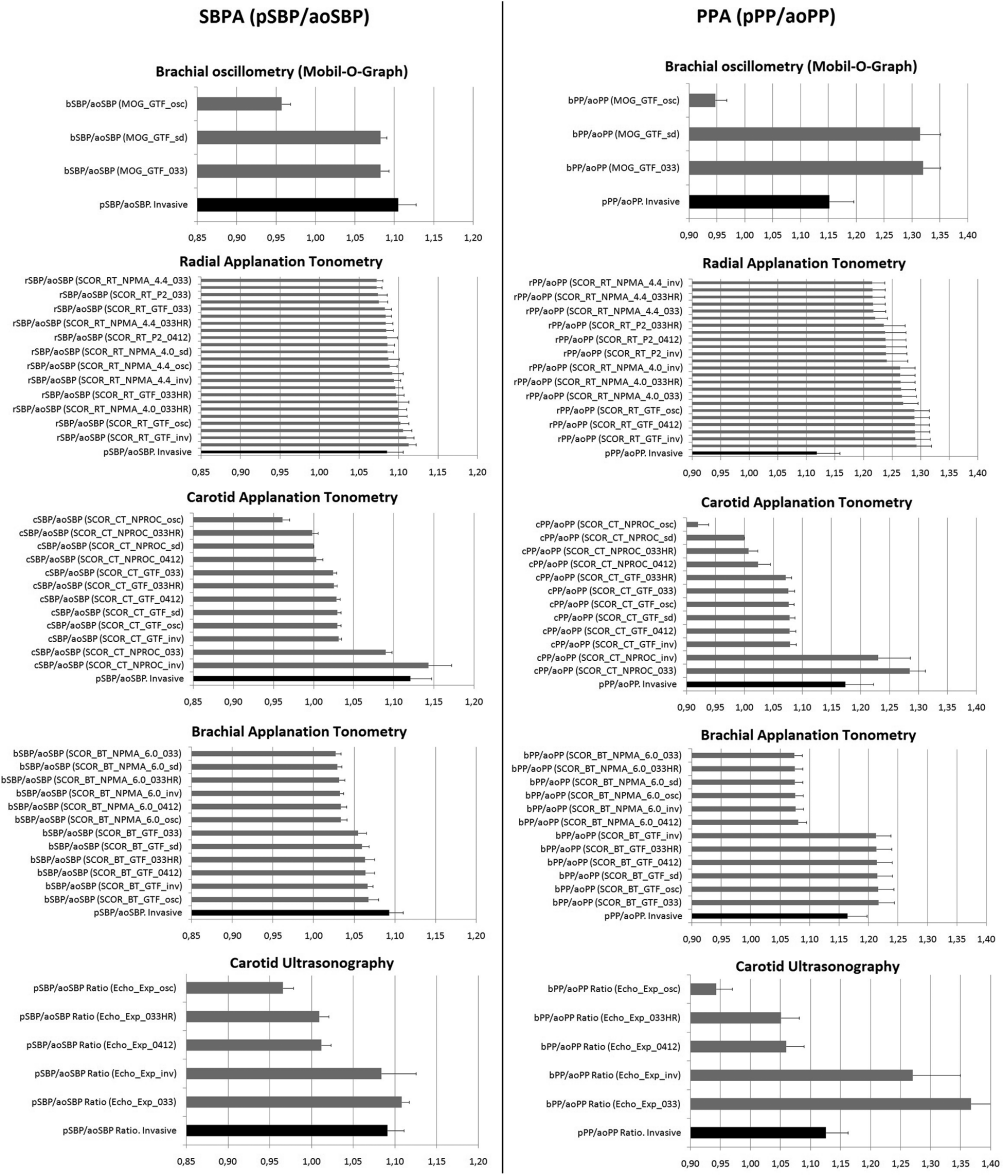
SBPA (pSBP/aoSBP) and PPA (pPP/aoPP) mean values (and 95%CI) obtained invasively and non-invasively. Black bar: invasive data obtained simultaneously (or immediately before or after) the corresponding non-invasive record. Note the scales (*x*-axis) are similar for both SBPA and PPA (e.g., 0.85–1.20 for SBPA, and 0.90–1.40 for PPA), which allows comparative analysis (vertically) of the graphs. Detailed quantitative information is in Supplementary File S1 and Tables S1–S5.

SBPA (SBPA = pSBP/aoSBP) and PPA (PPA = pPP/aoPP) were calculated from invasive and non-invasive aoSBP or aoPP data and from peripheral SBP and DBP (pSBP, pDBP; pPP = pSBP-pDBP) obtained at the BA (invasive and non-invasive recordings) or RA (Figure 2).

Two different approaches and devices were considered: one that allows obtaining different (new) peripheral BP values (e.g., SCOR) by using different schemes to calibrate the peripheral waveforms (a prior step to quantify aoBP) and another that does not (e.g., MOG). In this regard, the calibration of the SCOR-derived waveforms itself could result not only in different aoSBP and aoPP but also in new bSBP or radial SBP (rSBP) values. On the other hand, although MOG-derived bBP waveforms can be calibrated in three different ways to obtain the aoBP, the device does not give the new bSBP or bDBP values of the recalibrated waveforms, but only the bSBPosc and bDBPosc initially recorded are visible.

Finally, unlike methods using peripheral waveforms obtained from tonometry recordings that quantify aoBP after recalibration (resulting in new pSBP and pPP levels), when CCA waveforms (tonometry- or ultrasound-derived) are considered, the bSBP or bPP directly measured is used to calculate SBPA or PPA.

### 2.7. Variable names

The variables related to augmented pressure (AP) were identified with a sequence of words (separated by underscores), which allows how they were obtained to be identified (Figure 1):

(i) The parameter to which it refers (i.e., pSBP/aoSBP, pPP/aoPP)
(ii) The device used: “MOG,” “SCOR,” or “Echo” (Echography, ultrasound),
(iii) The arterial site/technique of recording: (i) CT or ultrasound, (ii) BA tonometry (BT), and RT,
(iv) The analysis considered: (i) “GTF” (use of a GTF), (ii) “NPMA” (use of the *N*-point moving average filter, indicating the filtering factor used in RA, “4.0” or “4.4,” and in BA, “6.0”), and (iii) “ExpAdj” (use of an exponential fit for the diameter-pressure transformation), and
(v) The calibration scheme of the recorded waveforms (“Inv,” “sd,” and “osc”) or the specific equation used to calibrate using the calculated bMBP (“033,” “033HR,” and “0412”).

To illustrate this, “pSBP/aoSBP (MOG_GTF_sd)” refers to the SBPA (“pSBP/aoSBP”) recorded with the “MOG” by applying a transfer function (“GTF”) and obtained when calibrating using the systo-diastolic method (“sd”).

### 2.8. Data and statistical analysis

**Figure 3:**
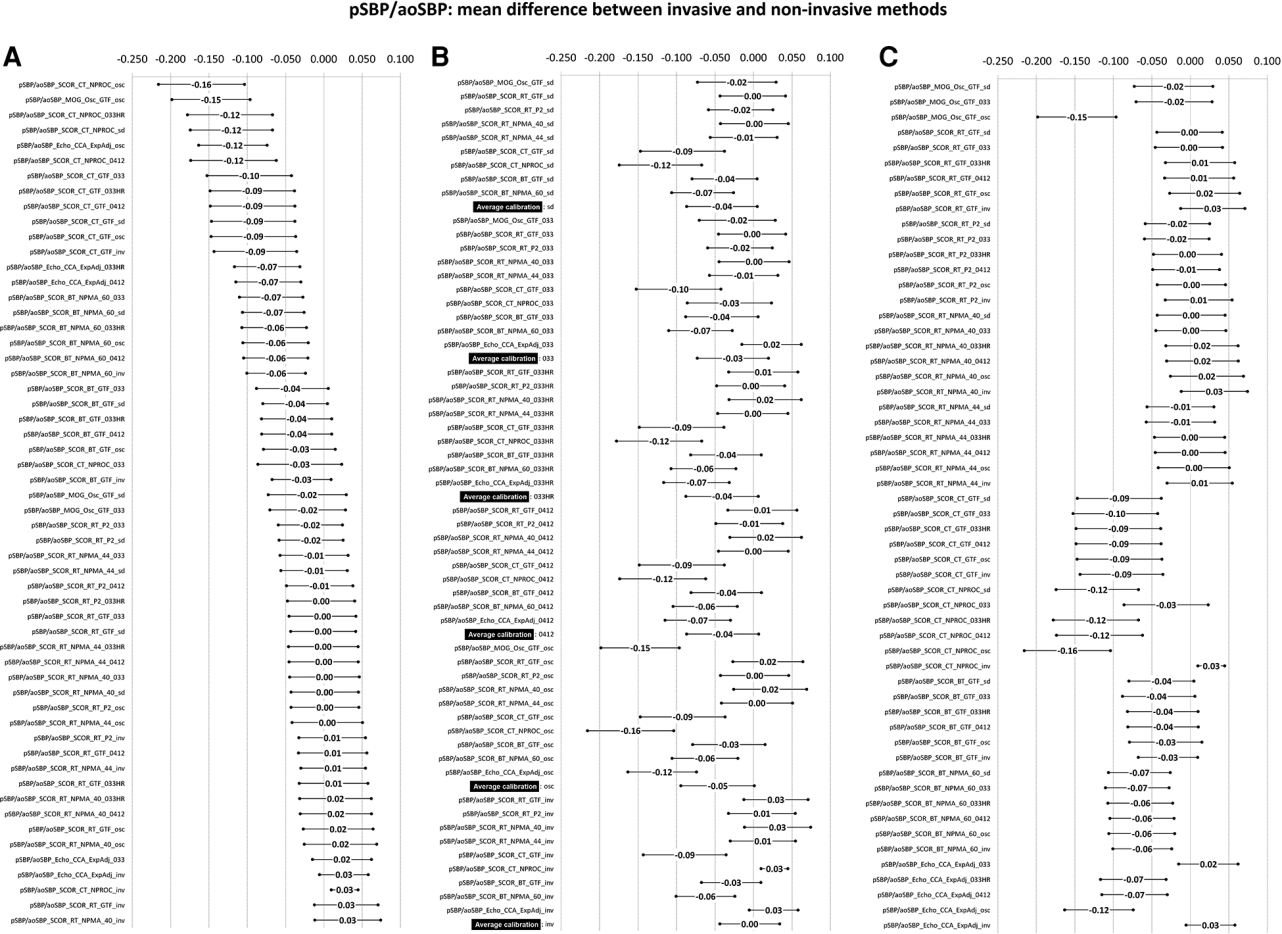
Bland–Altman derived mean error (and 95% CI) for comparisons between non-invasive and invasive SBPA (pSBP/aoSBP). Ordered according to error level (**A**), calibration scheme (**B**), and methodology (**C**). Detailed quantitative information is in Supplementary File S1 and Table S8.

**Figure 4:**
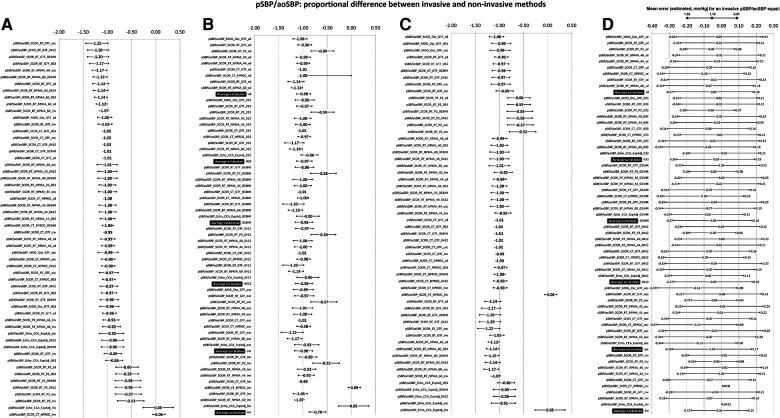
Bland–Altman proportional error (and 95% CI) for comparisons between non-invasive and invasive SBPA (pSBP/aoSBP) ordered according to error level (**A**), calibration scheme (**B**). and methodology (**C**). (**D**) Error plotted for three invasive SBPA levels: 0.87 (minimum), 1.10 (mean), and 1.33 (maximum). Detailed quantitative information: Supplementary File S1, Table S8.

**Figure 5:**
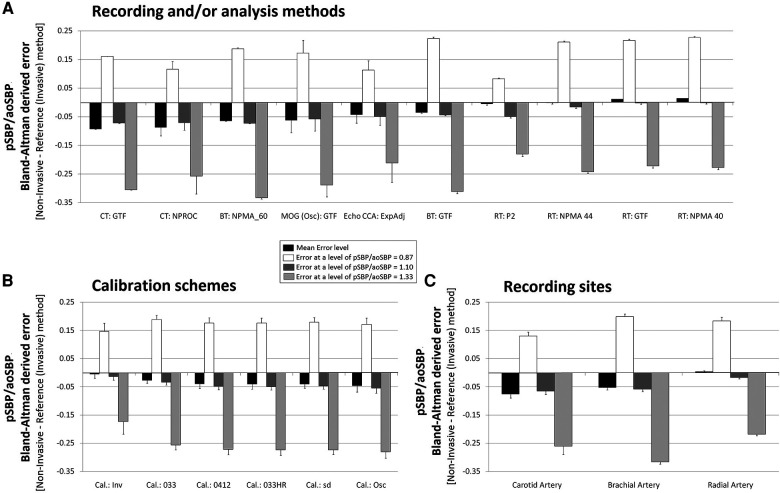
Pooled results for Bland–Altman derived mean errors obtained for comparisons between non-invasive and invasive SBPA (pSBP/aoSBP). The results are grouped according to recording and/or analysis method (**A**), calibration scheme (**B**), and recording site (**C**).

**Figure 6:**
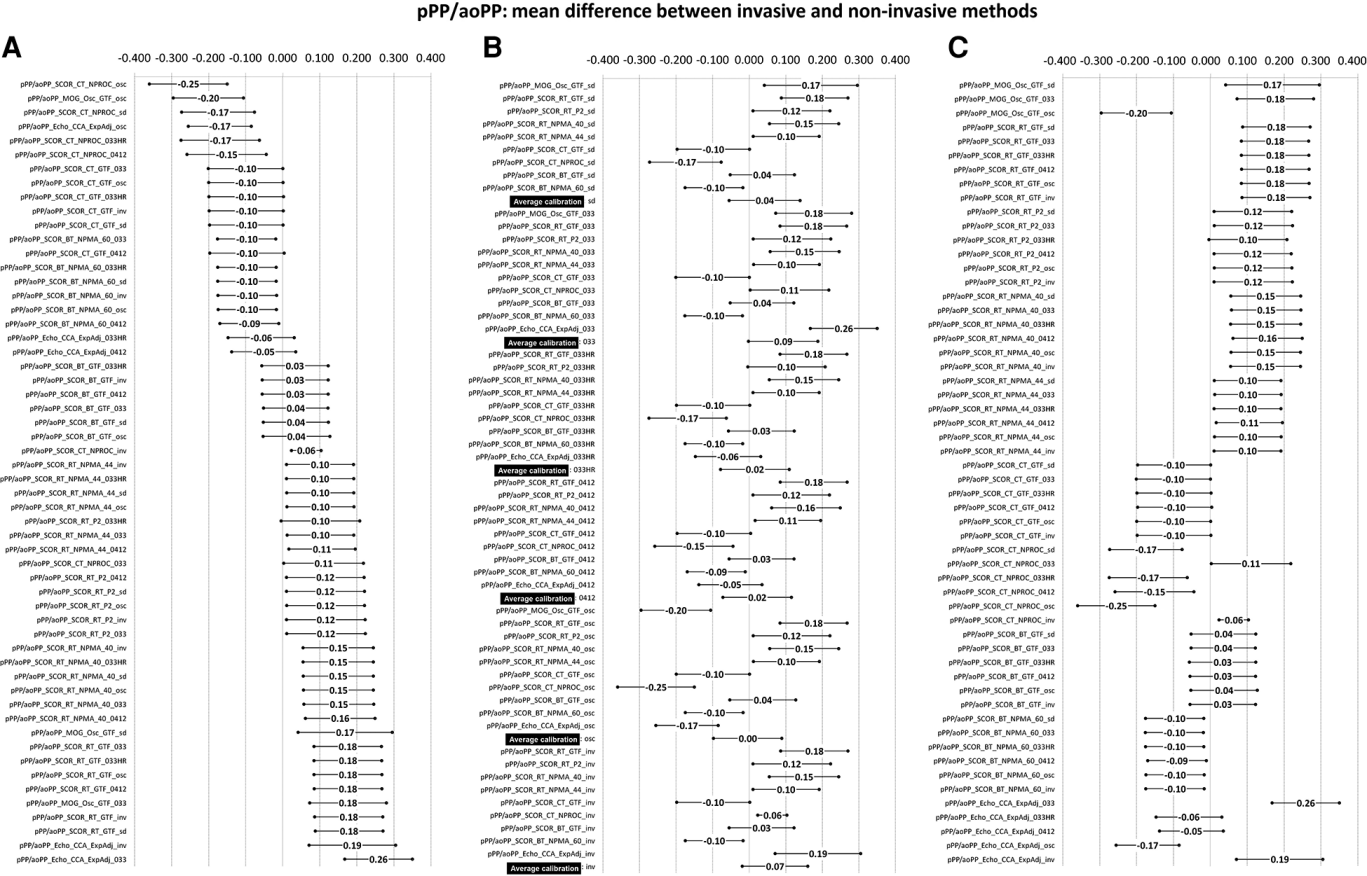
Bland–Altman derived mean error (and 95% CI) for comparisons between non-invasive and invasive PPA (pPP/aoPP) ordered according to error level (**A**), calibration scheme (**B**), and methodology (**C**). Detailed quantitative information is in Supplementary File S1 and Table S9.

**Figure 7:**
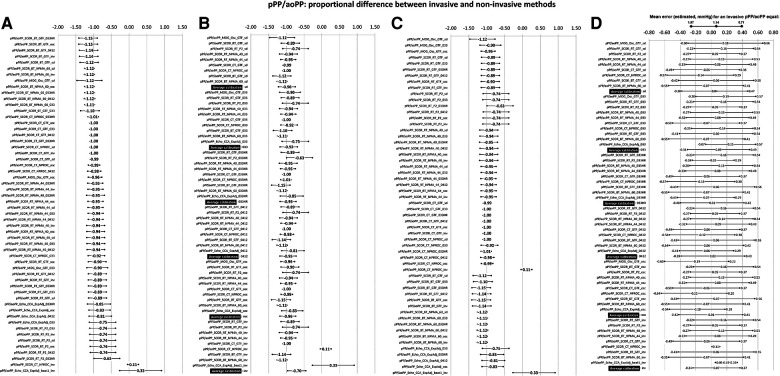
Bland–Altman derived proportional error (and 95% CI) for comparisons between non-invasive and invasive PPA (pPP/aoPP) ordered according to error level (**A**), calibration scheme (**B**), and methodology (**C**). (**D**) Error for three invasive PPA levels: 0.71 (minimum), 1.14 (mean), and 1.57 (maximum). Detailed quantitative information: Supplementary File S1 and Table S9.

**Figure 8:**
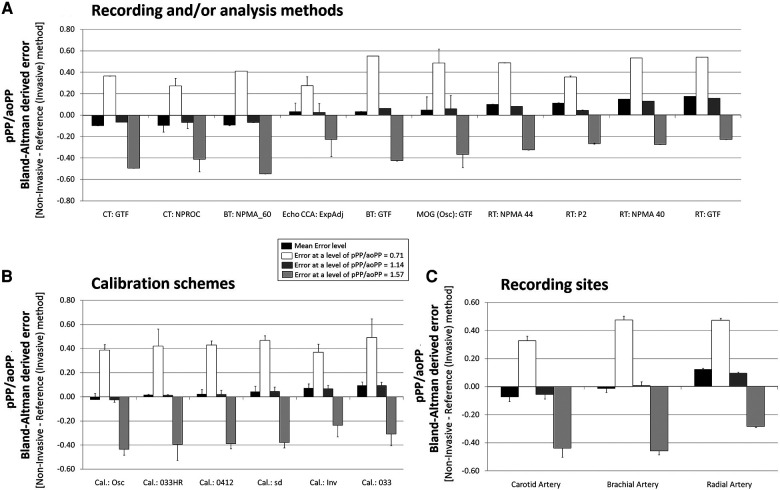
Pooled results for Bland–Altman derived mean errors obtained for comparisons between non-invasive and invasive PPA (pPP/aoPP). The results are grouped according to recording and/or analysis method (**A**), calibration scheme (**B**), and recording site (**C**).

Peripheral and central BP (invasive and non-invasive) levels obtained with the different approaches are shown in Supplementary File S1 and Tables S1–S5. After analyzing the characteristics of the group, and peripheral and central BP data obtained with the different methods (Tables 1, 2; Supplementary File S1, Tables S1–S5), we analyzed the correlation (association) and agreement between invasive and non-invasive SBPA and PPA data. Concordance correlation coefficient (CCC) [Supplementary File S1, Table S6 (for SBPA) and Table S7 (for PPA)] and the Bland–Altman analysis were considered (Supplementary File S1, Tables S8–S9). The Bland–Altman analysis enabled us to determine the mean and proportional errors between SBPA (Figures 3–5) and PPA (Figures 6–8) data, obtained with the invasive and non-invasive methods. In all cases, the Bland–Altman test corresponded to the reference method (invasive SBPA or PPA data, *x*-axis) against “non-invasive and invasive difference” (non-invasive minus invasive data, *y*-axis). For each Bland–Altman test, linear regression equations were quantified. Systematic error was considered present if the mean error was significantly different from 0 mmHg, and proportional error was considered present if the slope of the linear regression was statistically significant. Considering the mean and proportional errors obtained from the Bland–Altman test, the mean difference between invasive and non-invasive-derived SBPA and PPA was calculated (and graphed) for different SBPA (Figures 4, 5) and PPA (Figures 7, 8) values.

**Table 2:** Aortic and brachial invasive and brachial non-invasive blood pressure and heart rate levels.

Variable	MV	SE	SD	Min.	p25th	p50th	p75th	Max.	Range
Hemodynamic invasive records									
aoSBP invasive (mmHg)	**135**	4	23	77	122	134	154	179	102
aoMBP invasive (mmHg)	**94**	3	14	65	85	91	104	121	56
aoDBP invasive (mmHg)	**68**	2	10	52	62	65	75	92	40
aoPP invasive (mmHg)	**67**	4	20	22	55	64	82	103	81
HR invasive (beat/min)	**70**	3	14	49	56	68	78	104	55
bSBP invasive (mmHg)	**146**	5	28	77	133	144	168	189	112
bMBP invasive (mmHg)	**98**	3	15	66	89	98	111	122	57
bDBP invasive (mmHg)	**71**	2	10	54	63	70	80	92	38
bPP invasive (mmHg)	**75**	5	25	21	57	75	92	135	114
HR invasive (beat/min)	**70**	3	14	49	56	68	78	104	55
SBPA (bSBP/aoSBP) invasive	**1.08**	0.02	0.10	0.87	1.02	1.08	1.15	1.33	0.46
PPA (bPP/aoPP) invasive	**1.13**	0.03	0.19	0.71	1.02	1.15	1.23	1.57	0.87
Hemodynamic non-invasive records									
bSBP (oscillometry) (mmHg)	**137**	3	19	85	127	135	156	167	82
bDBP (oscillometry) (mmHg)	**81**	2	13	55	73	79	90	108	53
bPP (oscillometry) (mmHg)	**56**	3	14	29	45	55	67	91	62
HR (oscillometry) (mmHg)	**71**	3	14	44	59	72	81	105	61
bMBP_0.412_ (mmHg)	**104**	2	14	68	96	106	111	130	62
bMBP_0.33_ (mmHg)	**100**	2	13	65	92	100	106	125	60
bMBP_0.33HR_ (mmHg)	**104**	3	14	68	96	104	110	131	63
bMBP_osc_ (mmHg)	**107**	3	14	69	98	108	115	133	64

MV, mean value; SE and SD, standard error and deviation, respectively; Min. and Max., minimum and maximum value, respectively; p25th, p50th, and p75th, percentiles 25th, 50th, and 75th, respectively; SBP, MBP, DBP and PP, systolic, mean, diastolic, and pulse blood pressure, respectively. Prefix “b” and “ao” indicate brachial and aorta, respectively. HR, heart rate; SBPA and PPA, systolic and pulse pressure amplification, respectively. bMBP was obtained using oscillometry (osc) and three equations (see text).

Figure 5 (for SBPA) and Figure 8 (for PPA) show the pooled (aggregated) results (errors derived from the Bland–Altman analysis) when considering (i) the recording/analysis approach, (ii) the calibration method, and (iii) the arterial recording site.

According to the central limit theorem, considering the kurtosis and skewness distribution and the sample size (>25 subjects), a normal distribution was considered (51). Data analyses were performed with MedCalc (MedCalc Inc., Ostend, Belgium) and SPSS (IBM-SPSS Inc., Illinois, USA). A *p* < 0.05 was considered statistically significant.

## 3. Results

### 3.1. Clinical and hemodynamic characteristics

The studied subjects were mostly middle-aged (61 ± 19 years) men (59%) with BMI in the overweight range (27.2 ± 4.4 kg/m^2^). In addition, most patients had a history of hypertension (70%) and dyslipidemia (61%) (Table 1). The above is in agreement with the expectations for the subjects sent to invasive coronary evaluation.

Data about hemodynamic variables are detailed in Table 2 and Supplementary File S1 (Tables S1–S5). The group was characterized by a wide age (range: 14–89 years) and BP range. Invasive aoSBP values were 6.5% < 100 mmHg, 58.1% between 100 and 139 mmHg, 19.4% between 140 and 159 mmHg, and 16.1% ≥ 160 mmHg. In turn, invasive aoDBP values were 19.4% < 60 mmHg, 70.9% between 60 and 84 mmHg, and 9.7% > 85 mmHg.

### 3.2. SBPA and PPA levels

Figure 2 shows the SBPA and PPA values obtained from invasive (black bars) and non-invasive (gray bars) records. Similar scales were considered. Note the mean variability of the values. As expected, the SBPA and PPA values were generally above 1.0, although there were approaches that resulted in SBPA or PPA values below 1.0 (e.g., CCA_Echo_Exp_osc). In addition, note that for a given approach, the SBPA and PPA values were not the same.

### 3.3. Association between invasive and non-invasive SBPA and PPA

CCC levels obtained for the association between invasive and non-invasive SBPA and PPA varied depending on the technique, site, and calibration scheme considered. In this regard, CCC levels ranged between −0.1 and 0.94 for SBPA and −0.1 and 0.91 for PPA. The highest CCC level for SBPA (or PPA) was achieved when calibrating to invasive bBP (Supplementary File S1, Tables S6–S7). However, even when calibrating to invasive bBP, only pSBP/aoSBP_SCOR_TC_NPROC_inv (CCC = 0.94, *r* = 0.96) and pPP/aoPP_SCOR_TC_NPROC_inv (CCC = 0.91, *r* = 0.94) reached satisfactory CCC or Pearson’s coefficient values.

When considering non-invasive bBP levels for calibration, “pSBP/aoSBP_Echo_Exponential_beat1_033HR” (CCC = 0.27, *r* = 0.41) and “pPP/aoPP_P2_SCOR_RT_GTF_033HR” (CCC = 0.35, *r* = 0.37) showed the maximum CCC and Pearson’s coefficient values. Then, even the maximum values were very low. In turn, the lowest CCC level (e.g., for SBPA, minimum CCC = −0.1, mean CCC = 0.01, and maximum CCC = 0.24) was obtained when considering the “SD” calibration.

### 3.4. Agreement between invasive and non-invasive SBPA

The mean and proportional errors (bias) obtained when comparing invasive and non-invasive SBPA levels are detailed in Figures 3 and 4. In addition, Figure 5 shows pooled results of the mean errors when considering the (i) measuring and/analysis methodology, (ii) calibration method, and (iii) arterial measuring site.

The diverse approaches yielded a wide range of mean errors (−0.16 to 0.03). In general terms (61% = 34/56), the methods yielded negative errors. In 38% (21/56), the mean errors were statistically significant (Figure 3A; Supplementary File S1, Table S8). The pooled analysis of the records showed that overall, irrespective of the (i) recording method (Figure 5A), (ii) calibration method (Figure 5B), and (iii) recording site (Figure 5C), non-invasive measurements underestimated the SBPA levels.

#### 3.4.1. Invasive vs. non-invasive SBPA: mean and proportional error and analysis method

Disregarding the calibration scheme, we found that the pooled results showed that SBPA levels obtained from RA waveform analysis (tonometry) minimized the mean errors (Figure 5A). In turn, the different analyses done from RA waveforms (e.g., NPMA 40 or 44, GTF, and P2) did not show significant differences and did not show differences in mean error with respect to invasive SBPA (Figure 5A). On the other hand, SBPA analyses performed considering CCA waveforms (CT, GTF; CT, NPROC) showed the highest errors (Figure 5A).

Regardless of the calibration scheme and analyzing the interindividual error variability, we observed that SBPA data from RA records showed the greatest homogeneity in terms of error considering different SBPA levels (e.g., 0.87–1.33) (Figure 5A; Supplementary File S1, Table S8).

#### 3.4.2. Invasive vs. non-invasive SBPA: mean and proportional error and calibration

Regardless of the recording and/or analysis method, the calibration schemes resulted in lower non-invasive SBPA levels than invasive SBPA levels (negative mean errors) (Figure 5B). Overall, there were no major differences in mean errors when using different calibration schemes considering non-invasive bBP, but the lowest mean error was obtained when calibrating to “bMBP_033,_” and the highest mean error was obtained when calibrating to “osc.” The lowest errors were obtained when calibrating to invasive bBP levels (Figure 5B).

When proportional errors were analyzed, the results showed that the higher the invasive SBPA level, the greater the underestimation obtained with non-invasive approaches (Figure 5B).

#### 3.4.3. Invasive vs. non-invasive SBPA: mean and proportional error and recording site

In agreement with what has been already mentioned, non-invasive SBPA assessment based on RA recordings (followed by BA) allowed for minimizing mean errors with respect to invasive SBPA (regardless of the recording/analysis and calibration) (Figure 5C).

### 3.5. Agreement between invasive and non-invasive PPA

Figures 6 and 7 show the mean and proportional errors obtained when comparing invasive and non-invasive PPA. Figure 8 shows the Bland–Altman derived errors when considering the (i) recording and/analysis methods, (ii) calibration scheme, and (iii) recording site. The different approaches yielded a wide range of mean errors (−0.25 to 0.26). Most of the approaches (73%, 41/56) achieved significant mean errors (Figure 6).

#### 3.5.1. Invasive vs. non-invasive PPA: mean and proportional errors and analysis method

Regardless of the calibration scheme, PPA levels obtained from CCA ultrasound records and BA waveform analysis (BT, GTF; MOG, GTF) minimized the mean errors (Figure 8A). In contrast to the reported for SBPA, the analyses for PPA derived from RA waveforms resulted in PPA levels higher than those recorded invasively (positive mean error) (Figure 8A). The opposite was observed for CT:GTF and CT:NPROC registers.

When low and high PPA levels were considered (Figure 8A), all the approaches overestimated and underestimated PPA levels, respectively.

#### 3.5.2. Invasive vs. non-invasive PPA: mean and proportional error and calibration

Regardless of the recording and/or analysis method, the pooled results showed that the calibration schemes “osc,” “033HR,” and “0412” resulted in the lowest mean errors. The highest mean error was obtained with the “033” scheme (Figure 8B).

The higher the invasive PPA, the higher the underestimation observed with the non-invasive approaches, and vice versa (Figure 8B; Supplementary File S1, Table S9).

#### 3.5.3. Invasive vs. non-invasive PPA: mean and proportional error and recording site

Irrespective of the measuring method, pulse waveform analysis approach and/or calibration methods considered, PPA measurements based on BA-derived data allowed minimizing the error with respect to invasive PPA (Figure 8C).

## 4. Discussion

Despite substantial literature on SBPA and PPA, mainly aimed at assessing their capacity as markers of cardiovascular risk and identifying their determinants (e.g., arterial stiffness levels, age, and sex), there are surprisingly few articles (counted on the fingers of one hand) documenting the association and agreement between invasively and non-invasively measured SBPA or PPA levels. To our knowledge, this is the first study to investigate the level of association and agreement between invasive and non-invasive SBPA and PPA levels, using a wide variety of approaches (recording sites, signal processing methods, calibration schemes, and technologies).

### 4.1. Main results

The main results of this work can be summarized in seven points.

*First*, regardless of the calibration scheme and whether invasive or non-invasive bBP was used to calibrate, the CCC levels and Pearson’s coefficients obtained were extremely low, except for SBPA and PPA obtained from CT, without using a GTF. Then, it could be said that, in general, SBPA and PPA levels obtained with non-invasive approaches are not associated with the recorded invasively. The lack of association did not depend on the recording site, the analysis methodology, not on the calibration scheme, indicating that the limitation to achieving non-invasively SBPA and PPA levels that resemble those measured invasively is not just due to a single factor. This could be considered a “wake-up call” for all those who quantify SBPA or PPA for clinical and/or research purposes.

These low (or even non-existent) levels of strength of association were consistent with that obtained by Nakagomi et al. (25), in 45 patients in whom SBPA was measured invasively (catheterization, elective coronary angiography) and non-invasively (MOG), using two calibration schemes (C1, bSBP/bDBP, and C2, bDBP/bMBP). The authors found that non-invasively measured SBPA was associated with invasively measured SBPA only in C1 calibration (*r* = 0.33, *p* = 0.03) (25).

*Second*, when considering SBPA, the different non-invasive approaches led to (extremely) dissimilar results. On the other hand, in general, irrespective of the (i) recording method, (ii) calibration scheme, and (iii) recording site, non-invasive measurements underestimated SBPA. In addition, the error depended on the SBPA levels considered: the higher the invasive SBPA, the greater the underestimation (Figure 5B).

The existence of proportional error found in our work is consistent with that reported by Bui et al. (52). In this regard, these authors compared SBPA (calculated as “bSBP—aoSBP”) and PPA (calculated as “bPP—aoPP”) levels obtained (i) invasively (catheterization, fluid column pressure transducer) and (ii) non-invasively (brachial cuff-based method), using the “SphygmoCor Xcel” device (model EM4C, Atcor Medical, Sydney, Australia) and the BP Plus device (version 2, Uscom, Sydney, Australia). The waveforms of both non-invasive devices were calibrated with the corresponding cuff bSBP and bDBP measured by each device, respectively, and proprietary methods were automatically applied to estimate the central aoBP waveforms. The authors reported that, for both devices, the Bland–Altman plots revealed that non-invasive SBPA overestimated invasive SBPA at lower SBPA levels and underestimated it at higher SBPA levels. The results were similar for PPA (52).

Similar to us (when analyzing the whole group), these authors reported low agreement between cuff-based and invasive SBPA in all quartiles of invasive SBPA, especially in the lowest and highest quartiles, due to significantly lower individual variability of cuff-based SBPA than of invasive measures. As the authors themselves discussed, we can also state that our finding does not support the previously held assumption that type I central aoBP devices always provide appropriate estimates of SBPA (9). Clearly, work needs to be done to improve SBPA and PPA detection using non-invasive methods. Unfortunately, the authors mentioned that it was not possible to evaluate the results using other calibration methods, as the non-invasive devices they used did not allow them to do so (52).

*Third*, none of the different calibration schemes that considered non-invasive bBP to estimate SBPA were clearly better (ensuring lower mean error) than the others. However, in terms of overall “mean error,” the best approach seemed to be to calibrate with bMBP_033_.

*Fourth*, regardless of the calibration scheme, the pooled results showed that SBPA obtained from RA waveform analysis (applanation tonometry) minimized the mean errors (Figure 5A). In addition, SBPA levels derived from RA recordings showed the greatest homogeneity of bias when considering differences in the invasively recorded SBPA. In addition, there were no major differences in the results when applying different approaches to analyze RA waveforms (e.g., NPMA, GTF, P2) (Figure 5A). In contrast, SBPA data obtained from CCA waveforms showed the highest error (Figure 5A). Accordingly, a hierarchical order could be proposed to minimize the error in determining the SBPA: RA > BA > CCA. It is worth noting that in a recent work, we found that the lowest and highest mean errors in aoSBP estimation (non-invasive vs. invasive recordings) were achieved, respectively, from CCA and RA records (20). Thus, the best approaches to determine aoSBP and SBPA would differ.

This result shows that non-invasive estimation of SBPA (and PPA) is device- and technique-dependent and the results obtained with one technique do not apply to other devices. This is consistent with reports by other authors (52).

*Fifth*, when considering PPA levels, most approaches (73%, 41/56) achieved statistically significant mean errors (Figure 6). Compared to the observed for SBPA, the mean error levels when considering PPA were more homogeneously distributed, with approaches over- and underestimating invasive PPA. The higher the invasive PPA, the higher the underestimation obtained with the non-invasive approaches, and the opposite as well (existence of proportional error) (Figure 8B; Supplementary File S1, Table S9).

*Sixth*, in contrast to what was found for SBPA, when considering PPA regardless of the recording and/or analysis method, the pooled results showed that the calibration schemes “osc,” “033HR,” and “0412” ensured the lowest mean errors (Figure 8B), while the highest mean error level was obtained when calibrating with “033” (Figure 8B). Then, and surprisingly, the best calibration scheme when quantifying PPA could not be necessarily the best when quantifying SBPA.

*Seventh*, in contrast to what was reported for SBPA, regardless of the calibration scheme, the PPA levels obtained from CCA ultrasound recordings and BA waveform analysis (BT, GTF; MOG, and GTF) minimized the mean errors (Figure 8A). When quantifying PPA, data from RA waveforms resulted in PPA levels higher than those recorded invasively (positive mean error) (Figure 8A). The opposite was observed when considering CT:GTF and CT:NPROC recordings.

Altogether the findings above suggest that SBPA and PPA indices cannot be considered “synonymous.”

### 4.2. Methodological contribution

A significant part of our research work (and effort) has focused on identifying to what extent different non-invasive approaches that “in theory” enable us to assess the central (e.g., aortic) and/or peripheral (e.g., brachial) hemodynamics allow us to quantify intra-arterial BP levels. Recently, we focused on evaluating the level of agreement between aoSBP and aoPP recorded invasively and data obtained with more than 50 non-invasive approaches (20). To this end, simultaneous aoBP data were invasive and non-invasively obtained. However, at least in theory, an approach that allows an accurate non-invasive estimation of aoSBP or aoPP would not necessarily allow an adequate quantification of central-to-peripheral BP amplification (SBPA and PPA). In fact, in the “ARTERY Society task force consensus” (9), related to standardization of aoBP non-invasive records, measurement approaches (mainly devices) have been classified into two categories: (1) devices or systems that allow an adequate quantification of the (proportional) difference between aoBP and bBP (type I devices): in other words, such approaches allow an accurate assessment of the “degree of SBPA” enabling the monitoring of the effects of antihypertensive drugs whose responses may differ between bBP and aoBP (e.g., clinically relevant for hypertension management decisions); and (2) devices or systems that allow the derivation of accurate non-invasive aoSBP data (type II devices) (9): such approaches (type II devices) generally give aoSBP levels higher than bSBP (“reverse amplification”), which is likely to be non-physiological under normal (healthy) conditions and could be explained by the combination of underestimated bSBP (by the BA cuff), together with (in theory) the more accurate aoSBP estimation (9). Consequently, the more accurate non-invasive approach to quantify aoSBP or aoPP may be different from the more accurate method to quantify SBPA or PPA.

In this context, this study complements and deepens previous findings, focusing on the analysis of SBPA and PPA. To fulfill the aims of this work, it was necessary to measure not only invasive aoBP but also bBP (catheterization), as a way of knowing real central-to-peripheral BP amplification and being able to compare it with data obtained with several non-invasive approaches.

The results of this study add support to the hypothesis that the best approach (e.g., arterial recording site) for quantifying aoSBP (or aoPP) may not be the same for quantifying SBPA or PPA. For example, the findings show that regardless of the calibration scheme considered, the best approaches for determining aoSBP (or aoPP) were those based on CCA data (ultrasound or tonometry), followed by BA recordings since they resulted in aoSBP (or aoPP) values close to those recorded invasively (20). This was not the same for non-invasive SBPA and PPA quantification, since as stated above, regardless of the calibration scheme, the best approaches (ensuring lower mean errors) were those based on RA recordings and the least reliable were those based on CCA recordings. The above highlights the need for methodological accuracy and consensus to assess non-invasively SBPA (or PPA) and aoSBP (or aoPP). In addition, further research and development in this field is required.

### 4.3. Strengths and limitations

The work was not conducted in healthy subjects, which is common to most of this kind of studies taking into account the indications (and contraindications) of invasive studies. However, the subjects evaluated are representative of those for whom it is important to know these hemodynamic variables for decision-making in clinical practice.

The sample size could be considered “borderline” (moderate), but it should be noted that it was adequate to detect statistical differences and, consequently, achieved satisfactory statistical power. In this context, it should be noted that most of the studies similar to this one considered sample sizes smaller, equal, or slightly larger than those included in this work (19, 53). The invasive recordings in the BA, opposite to that of the limb used for the vascular access, and the second invasive recording at the aortic root were part of the research protocol and not of the catheterization for diagnostic purposes. The same consideration applies to all non-invasive BP recordings. Those measurements increased the duration of invasive evaluations by at least 30 min, which limited the number of volunteers consenting to participate in the study. In this context, the number of subjects included represents an important sample size for a study that aims to demonstrate the relevance of several issues, but not necessarily to be conclusive on this important topic, which necessarily requires further evaluations.

The differences between invasive and non-invasive SBPA or PPA levels could vary in association with factors like age, sex, HR, and vascular reactivity (54, 55). In fact, our results (Bland–Altman test) showed that there is a “proportional error” between invasively and non-invasively obtained central-to-peripheral amplification values. This indicates that the differences between invasive and non-invasive values depend on the existing levels of BP amplification in each person, which in turn depend on different variables (e.g., HR) (56–58). However, our sample was not enough large and/or heterogeneous enough to allow defining subgroups (e.g., considering the age, sex, and/or exposure of the subjects to risk factors) and to be able to apply statistical tests with sufficient power. Future, multicentre studies, allowing for the inclusion of a large number of subjects will be necessary to assess the impact of covariates or confounding factors on the results.

Fluid column BP transducers, rather than solid-state pressure sensors, were used. Clearly, solid-state sensors are characterized by a major accuracy in obtaining BP waveform, mainly because they can detect high-frequency components. However, fluid column transducers are widely used in clinical practice to obtain BP levels and are used in our University Hospital. It should be noted that the ARTERY Society Task Force Consensus Statement on protocol standardization stated that although micromanometer-tipped catheters are the preferred instruments, meticulously managed fluid column catheters may also be acceptable to accurately measure intra-arterial BP (8). In addition, in a systematic review and meta-analysis, it was reported that mean errors in the non-invasive estimation of aoSBP were similar when comparing fluid-filled and catheter-tipped transducers (19).

Finally, one issue to mention is that the invasive recordings in the ascending aorta and BA were not obtained simultaneously, but sequentially. However, the stability in HR and diastolic BP, and the subsequent similarity between the initial and final invasive aortic recordings, performed before and after invasive BA measurements, respectively, allowed to confirm hemodynamic stability.

## 5. Conclusions

Non-invasive measurements generally underestimated SBPA, and the higher the invasive SBPA, the higher the underestimation. When quantifying SBPA, there was no calibration scheme that was clearly better than the others. On the other hand, SBPA obtained from RA waveform analysis showed the minimum mean errors, whereas the highest ones were observed for data obtained from CCA waveforms.

The mean error levels observed when considering PPA were more homogeneously distributed. The higher the invasive PPA, the higher the underestimation obtained when using non-invasive approaches, and vice versa. There were differences in the calibration scheme. The “osc,” “033HR,” and “0412” ensured the lowest mean errors when PPA was quantified. In turn, PPA data obtained from CCA and BA waveform analyses minimized the mean errors.

In summary, altogether, the findings showed that (i) SBPA and PPA indices cannot be considered “synonymous” and (ii) non-invasive approaches would fail to accurately determine invasive SBPA or PPA levels, regardless of the recording site, analysis, and calibration methods.

## Data Availability

The raw data supporting the conclusions of this article will be made available by the authors, without undue reservation.

## Glossary

**Table:**

95%CI	95% confidence interval
aoBP	Central aortic blood pressure
aoDBP	Central aortic diastolic blood pressure
aoPP	Central aortic pulse pressure
aoSBP	Central aortic systolic blood pressure
BA	Brachial artery
bBP	Brachial artery blood pressure
bDBP	Brachial artery diastolic blood pressure
bDBPosc	Brachial artery diastolic blood pressure obtained with the oscillometric system
bMBP	Brachial artery mean blood pressure
bMBPosc	Brachial artery mean blood pressure obtained with the oscillometric system
BP	Blood pressure
bPP	Brachial artery pulse pressure
bPPosc	Brachial artery pulse pressure obtained with the oscillometric system
bSBP	Brachial artery systolic blood pressure
bSBPosc	Brachial artery systolic blood pressure obtained with the oscillometric system
BT	Brachial artery applanation tonometry
CCA	Common carotid artery
CCC	Lin’s concordance correlation coefficient
CT	Common carotid artery applanation tonometry
DBP	Diastolic blood pressure
Echo	Echography or vascular ultrasound
ExpAdj	Use of an exponential fit for the diameter-pressure transformation
GTF	Generalized transfer functions
HR	Heart rate
HRosc	Heart rate obtained with the oscillometric system (Mobil-O-Graph device)
MBP	Mean blood pressure
MOG	Mobil-O-Graph: oscillometry/plethysmography
NPMA	N-point moving average
NPROC	Not-processed approach´
osc	Calibration using oscillometric-measured bMBP
pDBP	Peripheral (brachial or radial) diastolic blood pressure
PP	Pulse pressure
PPA	Pulse pressure amplification
pPP	Peripheral (brachial or radial) pulse pressure
pSBP	Peripheral (brachial or radial) systolic blood pressure
RA	Radial artery
RT	Radial artery applanation tonometry
SBP	Systolic blood pressure
SBP2 or SBP2	Second peak or shoulder of the radial artery pulse waveform
SBPA	Systolic blood pressure amplification
SCOR	SphygmoCor: applanation tonometry
SD	Systo-diastolic calibration
